# ROX index performance to predict high-flow nasal oxygen outcome in Covid-19 related hypoxemic acute respiratory failure

**DOI:** 10.1186/s13613-023-01226-6

**Published:** 2024-01-18

**Authors:** Christophe Girault, Michael Bubenheim, Déborah Boyer, Pierre-Louis Declercq, Guillaume Schnell, Philippe Gouin, Jean-Baptiste Michot, Dorothée Carpentier, Steven Grangé, Gaëtan Béduneau, Fabienne Tamion

**Affiliations:** 1 Medical Intensive Care Unit, CHU Rouen, Normandie University, UNIROUEN, UR 3830, 76000 Rouen, France; 2 Department of Clinical Research and Innovation, CHU Rouen, 76000 Rouen, France; 3 Medical Intensive Care Unit, CHU Rouen, 76000 Rouen, France; 4 Medical and Surgical Intensive Care Unit, Dieppe Hospital, 76200 Dieppe, France; 5 Medical and Surgical Intensive Care Unit, Le Havre Hospital, 76600 Le Havre, France; 6 Department of Anesthesiology and Critical Care, CHU Rouen, 76000 Rouen, France; 7 Medical and Surgical Intensive Care Unit, Elbeuf Hospital, 76500 Elbeuf, France; 8 Medical Intensive Care Unit, CHU Rouen, Normandie University, UNIROUEN, Inserm, U1096, F-76000 Rouen, France; 9 Service de Médecine Intensive Et Réanimation, Hôpital Charles Nicolle, Centre Hospitalier Universitaire-Hôpitaux de Rouen, 37, Boulevard Gambetta, 76000 Cedex, France

**Keywords:** Acute respiratory failure, Covid-19 patients, High-flow nasal oxygen therapy, ROX index

## Abstract

**Background:**

Given the pathophysiology of hypoxemia in patients with Covid-19 acute respiratory failure (ARF), it seemed necessary to evaluate whether ROX index (ratio SpO_2_/FiO_2_ to respiratory rate) could accurately predict intubation or death in these patients initially treated by high-flow nasal oxygenation (HFNO). We aimed, therefore, to assess the accuracy of ROX index to discriminate between HFNO failure (sensitivity) and HFNO success (specificity).

**Methods:**

We designed a multicentre retrospective cohort study including consecutive patients with Covid-19 ARF. In addition to its accuracy, we assessed the usefulness of ROX index to predict HFNO failure (intubation or death) via logistic regression.

**Results:**

Among 218 ARF patients screened, 99 were first treated with HFNO, including 49 HFNO failures (46 intubations, 3 deaths before intubation). At HFNO initiation (H0), ROX index sensitivity was 63% (95%CI 48–77%) and specificity 76% (95%CI 62–87%) using Youden’s index. With 4.88 as ROX index cut-off at H12, sensitivity was 29% (95%CI 14–48%) and specificity 90% (95%CI 78–97%). Youden’s index yielded 8.73 as ROX index cut-off at H12, with 87% sensitivity (95%CI 70–96%) and 45% specificity (95%CI 31–60%). ROX index at H0 was associated with HFNO failure (*p* = 0.0005) in univariate analysis. Multivariate analysis showed that SAPS II (*p* = 0.0003) and radiographic extension of pulmonary injuries (*p* = 0.0263), rather than ROX index, were predictive of HFNO failure.

**Conclusions:**

ROX index cut-off values seem population-specific and the ROX index appears to have a technically acceptable but clinically low capability to discriminate between HFNO failures and successes in Covid-19 ARF patients. In addition, SAPS II and pulmonary injuries at ICU admission appear more useful than ROX index to predict the risk of intubation.

**Supplementary Information:**

The online version contains supplementary material available at 10.1186/s13613-023-01226-6.

## Introduction

During the worldwide Covid-19 pandemic, 15% to 20% of patients may need hospitalisation [1, 2]. Among them 5% to 10% require intensive care unit (ICU) admission for hypoxemic acute respiratory failure (ARF), and more than 50% of these severe Covid-19 ARF patients could develop acute respiratory distress syndrome (ARDS) [3].

Among different oxygenation strategies for ARF (standard or high-flow nasal oxygen therapy (HFNO), non-invasive (NIV) or invasive mechanical ventilation (MV)), HFNO has recently become the first-line treatment for hypoxemic ARF due to its physiological effects and potential benefit on patients’ outcomes [4–7]. Owing to a possible contamination of caregivers by SARS-CoV-2 aerosolization, HFNO was not recommended initially and rarely used in Covid-19 ARF [3, 8–10]. Later, increased knowledge and protective equipment for frontline healthcare workers [11] led to a wider HFNO use in this indication [12–15].

As with any non-invasive ventilatory management strategy, and although this has never been formally demonstrated in a prospective manner, HFNO should not delay, however, endotracheal intubation at the potential risk of worsening patients’ outcomes [16]. Thus, a simple index recorded at bedside could help clinicians to identify patients at high risk of HFNO failure and to start MV rapidly. For this purpose, ROX index, defined as the ratio of pulse oximetry/fraction of inspired oxygen relative to respiratory rate (SpO_2_/FiO_2_/RR), was proposed recently in “de novo” hypoxemic ARF [17–19].

Whilst HFNO allows to rapidly improve hypoxemia and decrease RR in patients if successful [5, 7], this benefit may be clinically less obvious in Covid-19 ARF patients. Indeed, because of different pathophysiological mechanisms of hypoxemia in these patients, they can be severely hypoxemic while being little or not tachypneic with relatively mild respiratory discomfort, the so-called “happy” or “silent” hypoxemia, especially at the initial phase of ARF [20, 21]. Therefore, the ROX index could be potentially less discriminant, with different cut-off values in patients with Covid-19 because of “silent hypoxemia” which is considered through the RR into the ROX index.

Consequently, we hypothesised that ROX index could be a potential useful diagnostic tool with sufficient accuracy in hypoxemic Covid-19 ARF patients. However, as suggested more recently [22–26], cut-off values for these patients may be different from those previously described for non-Covid-19 patients [17–19] and “silent hypoxemia” in Covid-19 patients [20, 21] could also impact the ROX index measurement. Therefore, we aimed to evaluate the accuracy and usefulness of ROX index to predict the risk of intubation or death in Covid-19 ARF patients initially treated with HFNO in ICU.

## Methods

### Study design

We conducted a multicentre cohort study during the first Covid-19 outbreak in Eastern Normandy between March 10th and May 25th, 2020. The local ethics committee approved the study (approval number E2020-40). Due to its retrospective observational design, patients’ written informed consent was not required.

### Study population and HFNO strategy

We screened all consecutive adult patients referred to five ICUs, i.e., two in Rouen University hospital and three in regional community hospitals (Elbeuf, Dieppe, Le Havre), and admitted for hypoxemic ARF (need for standard O_2_ ≥ 6 L/min to reach a pulse oximetry (SpO_2_) ≥ 92%, with or without clinical signs of respiratory distress). For inclusion, patients had to have highly suspected or confirmed SARS-CoV-2 pneumonia based on CT-scan imaging and/or RT-PCR, and had to be treated with HFNO as first-line respiratory support in ICU. We excluded Covid-19 ARF patients transferred from other French regions, patients treated only with standard O_2_ < 6 L/min, and patients requiring immediate intubation or with do-not-intubate order at ICU admission. All patients were followed until hospital discharge or death, whichever occurred first. Some patients from Rouen University ICUs were included in a previously published study [15].

Depending on participating ICUs, HFNO was delivered either by Optiflow^®^ (Fisher & Paykel Healthcare, Auckland, New Zealand) or Airvo2^®^ (Fisher & Paykel Healthcare, Auckland, New Zealand) device, or through an ICU ventilator. As previously described, initial HFNO settings were the highest tolerated humidified flow rate ranging from 30 to 60L/min, and a FiO_2_ titrated for a SpO_2_ > 92% [6].

### Evaluation criteria and data collection

The primary objective was to assess the accuracy of ROX index, hence we determined a cut-off value to discriminate between HFNO failure (sensitivity) and HFNO success (specificity). More precisely, HFNO failure was defined as the need for intubation or death before intubation within 28 days after ICU admission and HFNO initiation (H0), since NIV is not usually used as second-line ventilatory support in hypoxemic ARF in participating ICUs [6]. The final decision for intubation was made by the attending ICU physician, the main criteria being shared by all participating ICUs [6]. To estimate ROX index specificity, patients who did not need MV and who did not die within 28 days after H0 were classified as HFNO success. The compromise between late intubation (sensitivity) and avoiding MV (specificity) was established at H0, as well as at prominent time-points thereafter [17, 18].

To reach our secondary objective to predict HFNO failure within 28 days after H0, we retrieved the following information from patients’ electronic files: patients’ characteristics (age, sex, body mass index, comorbidities), Simplified Acute Physiologic Score II (SAPS II) [27] and Sequential Organ Failure Assessment (SOFA) [28], Covid-19 history (symptom onset, RT-PCR assay result, type and pulmonary extension of chest-X ray or CT-scan injuries), renal or hemodynamic organ failure, clinical respiratory conditions and parameters (interface and flow rate with standard O_2_, estimated FiO_2_ [29], SpO_2_, RR), arterial blood gas (ABG) at ICU admission before HFNO initiation. During the first 24 h after HFNO initiation, clinical respiratory conditions and parameters (HFNC flow rate, FiO_2_, SpO_2_, RR) were recorded at H0, H2-H4, H6, H12, H18, H24, as well as ABG at H2-H4, H6-H12 and H12-H24.

Furthermore, we collected data regarding time, cause and duration of intubation, respiratory conditions at ICU discharge, vital status and date of ICU and hospital discharge.

### Statistical analysis

To assess ROX index accuracy, the primary endpoint was sensitivity based on the cut-off of 4.88 at H12 under HFNO as proposed previously by Roca et al. [17, 18] in non-Covid-19 ARF patients. For this purpose, sample size was determined such that the half-width of the 95% confidence interval for sensitivity should not exceed 20%. A cohort rather than a case–control design was chosen because knowledge of prevalence is needed for prediction of HFNO failure during the first 28 days after ICU admission. The receiver operating characteristic (ROC) curve visualizes ROX index accuracy. To this end, specificity for a given time-point was estimated by considering those patients as controls who received HFNO at this time and had no HFNO failure before hospital discharge or day 28 after ICU admission, whichever came first. Sensitivity was estimated correspondingly, i.e., patients who received HFNO at the given time-point and had HFNO failure subsequently. Youden’s index served to determine the cut-off, defined as the largest difference between sensitivity and 1-specificity.

To reach the second objective, the cumulative incidence function of HFNO failure was depicted because, unlike those patients who decease, not all patients need intubation. Then covariates, fixed at the time prediction starts, were screened one by one to assess their influence on the risk of HFNO failure within the first 28 days after ICU admission using logistic regression. Covariates significantly related to this risk were considered for multivariable analysis and backward selection was used to establish a parsimonious model.

Patient characteristics of at least ordinal level were described using the median with the first (Q1) and third quartile (Q3). The Kruskall–Wallis’s test was used to come to know whether there was a potential center effect. Freeman–Halton’s test and Wilcoxon’s test for independent samples were used to compare patients with to those without vasopressor at ICU admission.

Given the study’s exploratory nature, no correction for multiple testing was carried out and a p-value less than 0.05 was considered statistically significant. Analyses were performed using SAS software from SAS Institute Inc. (Cary, NC, USA).

## Results

**Fig. 1:**
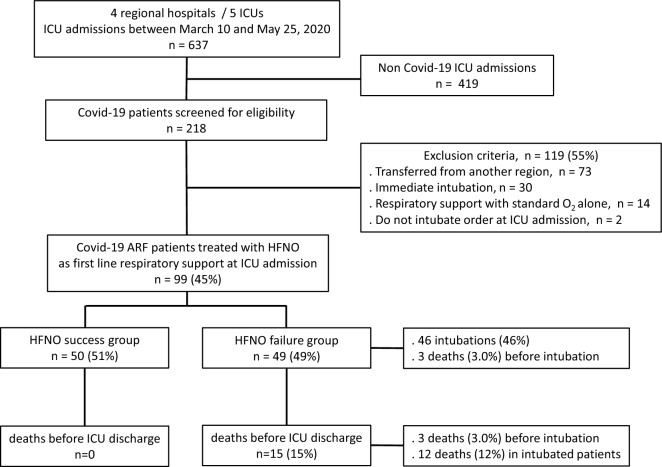
Flowchart of the study and HFNO outcome *ICU* intensive care unit, *ARF* acute respiratory failure, *HFNO* high-flow nasal oxygen therapy

Among 218 Covid-19 hypoxemic ARF patients admitted to ICUs, 99 (45%) were treated with HFNO alone as first-line respiratory support (Fig. 1). During the 28 days following ICU admission, 50 patients (51%) remained alive without requiring intubation (HFNO success group) whereas 49 patients (49%) failed (HFNO failure group), including 3 patients who died without prior intubation and 46 who were intubated. Among the latter, 12 died before ICU discharge (Fig. 1). Table 1 shows patients’ characteristics at ICU admission and HFNO initiation for the overall population and according to HFNO success and failure groups.

**Table 1:** Patients’ characteristics and conditions at ICU admission and at discharge according to HFNO outcome

Parameters	Overall population	HFNO success group	HFNO failure group	p-value*
	*n* = 99	*n* = 50	*n* = 49	
**Characteristics at ICU admission**				
Age (years)	67 (54–73)	61 (51–72)	68 (61–73)	0.0592
Sex M/F, n (%)	66/33 (67%/33%)	31/19 (62%/38%)	35/14 (71%/29%)	0.3210
BMI (kg/m^2^)	28.0 (25.2–32.0)	28.1 (25.3–32.4)	27.8 (24.8–30.4)	0.2676
Comorbidities,* n* (%)				
Diabetes mellitus	29 (29%)	12 (24%)	17 (35%)	0.2445
Hypertension	58 (59%)	30 (60%)	28 (57%)	0.7730
Underlying cardiac disease	17 (17%)	6 (12%)	11 (22%)	0.1740
Underlying respiratory disease	22 (22%)	11 (22%)	11 (22%)	0.9572
Chronic renal failure	6 (6%)	3 (6%)	3 (6%)	0.9796
Chronic liver disease	0	0	0	–
Immunosuppression	14 (14%)	9 (18%)	5 (10%)	0.2713
Other	30 (30%)	14 (28%)	16 (33%)	0.6147
SAPS II	35 (26–43)	29 (23–36)	41 (34–49)	** < 0.0001**
SOFA score	2 (2–4)	2 (2–3)	4 (2–5)	**0.0027**
Acute renal failure,* n* (%)	14 (14%)	3 (6%)	11 (22%)	**0.0277**
Hemodynamic failure,* n* (%)	12 (12%)	0	12 (24%)	< 0.0001**
Covid-19 history				
Time since symptom onset (days)	9 (7–13)	9 (7–12)	8 (7–14)	0.7381
Hospital stay before ICU admission (days)	1 (0–3)	1 (0–3)	1 (0–3)	0.3852
Chest-X ray and/or CT-scan findings,* n* (%)				
Type of pulmonary injuries				
Ground-glass opacities	89 (90%)	46 (92%)	43 (88%)	0.7183
Crazy-paving	49 (49%)	29 (58%)	20 (41%)	0.1545
Consolidation	64 (64%)	36 (72%)	28 (57%)	0.2444
Unknown	5 (5%)	1 (2%)	4 (8%)	-
Severity of pulmonary injuries, n (%)				
No. of affected quadrants	4 (3–4)	3 (2–4)	4 (3–4)	**0.0119**
Extension of injuries on chest X-ray or CT,* n* (%)				**0.0067**
< 25%	32 (32%)	25 (50%)	7 (14%)	
25–50%,	30 (30%)	12 (24%)	18 (37%)	-
50–75%	23 (23%)	10 (20%)	13 (27%)	-
> 75%	8 (8%)	2 (4%)	6 (12%)	-
Unknown	6 (6%)	1 (2%)	5 (10%)	-
Associated pulmonary embolism, n (%)	11 (11%)	5 (10%)	6 (12%)	0.6382
**Respiratory conditions at HFNO initiation**				
Time between ICU admission and HFNO initiation (hours)	0.1 (0–1.4)	0.1 (0–1.0)	0.1 (0–2.0)	0.9220
Flow rate (L/min)	50 (30–50)	45 (40–50)	50 (30–50)	0.2724
FiO_2_ (%)	60 (50–70)	50 (40–60)	60 (50–80)	**0.0006**
SpO_2_ (%)	95 (93–97)	96 (94–97)	94 (92–96)	**0.0011**
SpO_2_/FiO_2_ (%)	163 (137–196)	192 (158–238)	155 (119–186)	**0.0004**
RR (cycles/min)	25 (20–29)	24 (18–28)	25 (22–30)	0.3555
Rox index (SpO_2_/FiO_2_/RR)	6.71 (5.27–9.50)	7.92 (6.23–10.33)	5.64 (4.57–7.37)	**0.0005**
Arterial blood gas before HFNO initiation (n = 89)				
PaO_2_ (mmHg)	70 (62–83)	78 (67–86)	63 (57–73)	**0.0132**
PaO_2_/FiO_2_ (mmHg)	146 (117–181)	160 (131–204)	136 (109–163)	**0.0087**
SaO_2_ (%)	95 (93–97)	96 (94–98)	94 (91–96)	**0.0157**
pH	7.45 (7.42–7.47)	7.45 (7.43–7.48)	7.45 (7.41–7.47)	0.6256
PaCO_2_ (mmHg)	34 (32–40)	35 (32–41)	34 (32–38)	0.2537
HCO3^−^ (mmol/L)	23.6 (21.7–25.9)	23.6 (22.0–26.7)	23.5 (21.1–25.4)	0.3928
**Conditions at discharge**				
Intubation,* n* (%)	46 (46%)	-	46 (94%)	-
Death before intubation,* n* (%)	3 (3%)	-	3 (6%)	-
HFNO duration (hours)	54 (22–147)	136 (49–183)	28 (11–61)	< 0.0001***
Standard O_2_ at ICU discharge among survivors,* n* (%)	63 (75%)	41 (82%)	22 (65%)	0.1222**
Flow rate (L/min)	2 (1–3)	3 (2–3)	2 (1–3)	0.0184***
ICU mortality at day 28 after ICU admission,* n* (%)	13 (13%)	0	13 (27%)	< 0.0001**
ICU length of stay (days)	11 (6 -19)	7 (5 -11)	19 (12 -27)	< 0.0001***
ICU mortality,* n* (%)	15 (15%)	0	15 (31%)	< 0.0001**
Hospital length of stay (days)	21 (13–36)	18 (12–25)	31 (15–54)	0.0025***
Hospital mortality after ICU discharge,* n* (%)	0	0	0	-

Bold values depict significant statistical values

*HFNO* high-flow nasal oxygen therapy, *BMI* body mass index, *SAPS* Simplified Acute Physiology Score, *SOFA* Sequential Organ Failure Assessment; *FiO*_*2*_ fraction of inspired oxygen, *SpO*_*2*_ pulse oxygen saturation, *RR* respiratory rate, *ICU* intensive care unit; values are expressed as n (%) or median (Q1-Q3); * = logistic regression unless stated otherwise; ** = Fisher’s exact test; *** = Wilcoxon-Mann–Whitney test

HFNO failure group comprises 46 intubations and 3 deaths before intubation

**Fig. 2:**
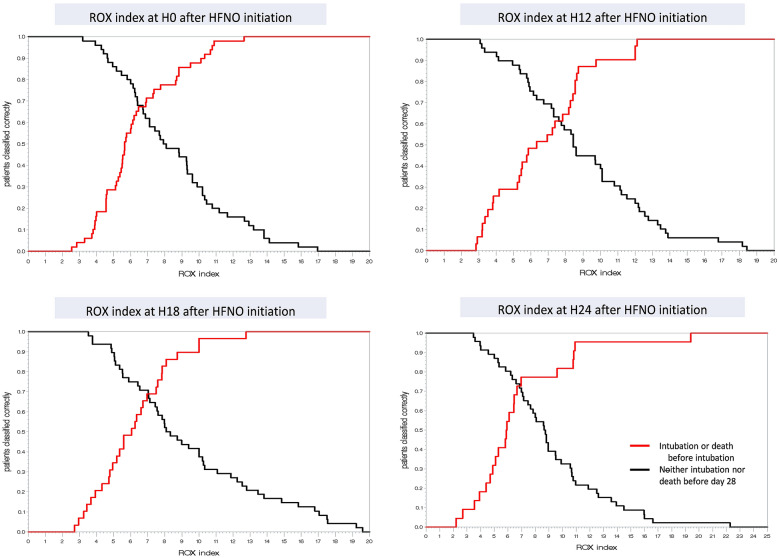
ROX index performance to predict HFNO failure at different times after HFNO initiation HFNO: high-flow nasal oxygen therapy; red line gives proportion of patients in the HFNO failure group with a ROX index ≤ a chosen cut-off value; black line gives proportion of patients in the HFNO success group with a ROX index ≤ a chosen cut-off value. For example: at H12, using a Rox index of ≤ 9.75 as cut-off would identify 90% of patients with HFNO failure after H12, whereas this cut-off would identify only 41% of patients with HFNO success after H12, avoiding intubation

As regards ROX index accuracy at H0, the area under the ROC curve (AUROC) was 0.70, representing a relative acceptable discrimination (see Additional file 1: Figure S1), and Youden’s index yielded 6.20 as cut-off value (Table 2). For this cut-off, sensitivity was 63% [95%CI 48%-77%] and specificity was 76% [95%CI 62–87%]. Hence, among the 49 patients who failed HFNO (intubation or death) before day 28, 31 (63%) had a ROX index ≤ 6.20 at H0 and were correctly classified positive. Among the 50 patients who remained alive without intubation, 12 (24%) had a ROX index ≤ 6.20 and were hence classified falsely positive (Fig. 2 and see Additional file 1: Figure S2).

**Table 2:** ROX index accuracy to predict HFNO failure at different times after HFNO initiation

Time after HFNO initiation	Maximum of Youden’s index	Rox index cut-off	Sensitivity*	95% CI limits for sensitivity		Specificity	95% CI limits for specificity		AUROC
				Lower	Upper		Lower	Upper	
H0	0.393	6.20	63.3%	48.3%	76.6%	76.0%	61.8%	86.9%	0.70
H2-H4	0.413	6.88	78.0%	62.4%	89.4%	63.3%	48.3%	76.6%	0.71
H6	0.361	7.00	75.7%	58.8%	88.2%	60.4%	45.3%	74.2%	0.68
H12	0.320	8.73	87.1%	70.2%	96.4%	44.9%	30.7%	59.8%	0.66
H18	0.398	6.96	69.0%	49.2%	84.7%	70.8%	55.9%	83.0%	0.70
H24	0.490	6.96	77.3%	54.6%	92.2%	71.7%	56.5%	84.0%	0.75
H48	0.343	5.00	43.8%	19.8%	70.1%	90.5%	77.4%	97.3%	0.67
H72	0.543	5.78	80.0%	44.4%	97.5%	74.3%	56.7%	87.5%	0.77

*HFNO* high-flow nasal oxygen therapy, *AUROC* area under the receiver operating characteristic curve, *CI* confidence interval; * = proportion of patients with ROX index ≤ cut-off among those who failed HFNO within 28 days after ICU admission

Also, at H2–H4, H18 and H24, ROX index accuracy was still relatively acceptable (0.70 ≤ AUROC < 0.80) to identify patients who needed intubation (Table 2). Until H12, both the ROX index cut-off value and its sensitivity increased, whereas specificity decreased. At H12, the cut-off based on Youden’s index was 8.73, yielding a sensitivity of 87% [95%CI 70–96%] and a specificity of 45% [95%CI 31–60%] (Table 2). Hence, though identifying 87% of subsequent HFNO failures, a ROX index ≤ 8.73 at H12 was also observed for the majority (55%) of patients who did not need intubation. When applying the previously suggested ROX index cut-off value of 4.88 [17, 18] to our population at H12, sensitivity was 29% [95%CI 14–48%] with a specificity of 90% [95%CI 78-97%].

After H24, when no more than 25 patients failed (see Additional file 1: Figure S3), confidence limits for sensitivity were wide and even < 50% (Table 2) suggesting the absence of a reliable basis for appreciating ROX-index discrimination power.

As regards patients with HFNO failure, conditions of intubation and clinical respiratory parameters are shown in the Additional file 1: Table S4. The main cause of intubation was Covid-19 ARF impairment (91%) and occurred within 29 h after ICU admission for half of them. Intubated patients were severely hypoxemic with a median PaO_2_/FiO_2_ of 73 mmHg and a ROX index of 3.83 at the time of intubation. Of note, 45/49 HFNO failures (92%) occurred within 4 days after HFNO initiation (see Additional file 1: Figure S3). We also analyzed intubated patients by underlying hemodynamic status (vasopressors vs non vasopressors) at ICU admission, which is recognized as a risk factor of HFNO failure but not taken into account in the ROX index. Intubated patients with vasopressors were more rapidly intubated after ICU admission (*p* = 0.0017) and HFNO initiation (*p* = 0.0010), as well as they had lower PaCO_2_ levels (*p* = 0.0262) at the time of intubation as compared to intubated patients without vasopressor at ICU admission (see Additional file 1: Table S4).

By screening covariates at H0 (univariate analysis), potential predictive factors for HFNO failure were (Table 1): SAPSII (*p* < 0.0001), SOFA score (*p* = 0.0027), presence of acute renal failure (*p* = 0.0277), hemodynamic failure (*p* < 0.0001) and severity of Covid-19 pulmonary lesions, in terms of number of affected quadrants (p = 0.0119) and injury extension (*p* = 0.0067). As regards respiratory conditions at H0, risk factors associated with HFNO failure were (Table 1): FiO_2_ (*p* = 0.0006), SpO_2_ (*p* = 0.0011), SpO_2_ /FiO_2_ (*p* = 0.0004), and ROX index (*p* = 0.0005). By contrast, no evidence was found of RR being related to HFNO failure (*p* = 0.3555). In patients with available ABG before HFNO initiation (*n* = 89), we found PaO_2_ (*p* = 0.0132), SaO_2_ (*p* = 0.0157) and PaO_2_/FiO_2_ ratio (*p* = 0.0087) related to a risk of HFNO failure.

Using backward selection method, the multivariate analysis yielded the following factors known at H0, which were predictive for HFNO failure within 28 days after ICU admission: SAPSII (*p* = 0.0003), PaO_2_ (*p* = 0.0497) and radiographic extension of pulmonary injuries (*p* = 0.0263) (Table 3).

**Table 3:** Risk factors at HFNO initiation to predict HFNO failure according to respiratory parameters (multivariate analysis)

Retained covariates known at HFNO initiation	Patients (*n*)	OR (95% CI)	*p*-value (level)	*p*-value (variable)
SAPS II (per additional point)	83	1.19 (1.08–1.30)		**0.0003**
PaO_2_ (per additional mmHg)	83	0.96 (0.92–1.00)		**0.0497**
Extension of pulmonary injuries on chest X-ray or CT				**0.0263**
< 25%	28	Ref		
25–50%	28	10.32 (1.91–55.67)	**0.0067**	
50–75%	21	7.96 (1.55–41.01)	**0.0131**	
> 75%	6	9.75 (0.81–117.85)	0.0732	

Bold values depict significant statistical values

*HFNO* high-flow nasal oxygen therapy, *SAPS* Simplified Acute Physiology Score, *OR* odds-ratio, *CI* confidence interval, *ref * reference category for level comparisons via OR

Regarding HFNO outcomes (Table 1), failure occurred 28 h after H0 on the median, whereas patients in the success group used HFNO for 136 h (*p* < 0.0001). ICU (*p* < 0.0001) and hospital (*p* = 0.0025) length of stay were longer among HFNO failures compared to HFNO successes. Deaths occurred among HFNO failures only, with a 28-day ICU mortality of 27% (*p* < 0.0001) and no death occurred in hospital after ICU discharge (Table 1).

In particular, there was no evidence of center effects with respect to HFNO outcome, ROX index at H0, and, among those who were intubated, HFNO duration before intubation (see Additional file 1: Table S5).

## Discussion

Our results show that, after HFNO initiation, the ROX index discriminates only in a technically acceptable manner at different time-points between patients who were intubated or died within 28 days after ICU admission, and patients who did not need intubation. It also highlights that population- and setting-specific cut-off values rather than defaults like those previously suggested [17–19] should be used, in particular in hypoxemic ARF patients with Covid-19. Finally, we also identified SAPS II and the severity of pulmonary lesions at ICU admission as potential independent risk factors for HFNO failure.

Due to its relevant physiological effects and potential benefit on patients’ outcomes [4–7], and although not recommended at the beginning of the SARS-CoV-2 pandemic [3, 8–10], more and more Covid-19 ARF patients were managed with HFNO [12–15]. In our study, the intubation and ICU mortality rates were 49% and 15%, respectively. Previous studies reported intubation rates varying from 18 to 56%, and ICU or 28-day mortality rates ranging from 7 to 25% in this population [12–15].

Nevertheless, timely intubation remains a major challenge for ICU clinicians when managing severe hypoxemic ARF patients, including those treated with HFNO [16, 30, 31]. In this way, ROX index was found useful in non-Covid-19 ARF patients [17, 18]. A ROX index ≥ 4.88 at H2, H6 and H12 after HFNO initiation predicted a lower risk of intubation, with accuracy increasing over time [17, 18]. More recently, ROX index was evaluated in Covid-19 ARF populations admitted to ICU [22–26]. Different ROX index cut-off values were proposed to discriminate best between patients on HFNO needing intubation or not, not only in non-Covid-19 [17–19] but also in Covid-19 ARF populations [22–26]. Similar to our study, higher ROX cut-offs than 4.88 were generally reported to prevent late intubation in Covid-19 ARF populations [22–26]. For instance, in our study, a cut-off of 4.88 to prevent late intubation would have identified only a minority of those who failed after H12 (29%), whereas a vast majority (87%) would have been identified with the cut-off based on Youden’s index.

Differences regarding cut-off values might be explained, at least partially, by the pathophysiological mechanisms of “happy” or “silent” hypoxemia which can be difficult to recognize early and hence delay ARF management in Covid-19 patients [20, 21]. Indeed, patients can be severely hypoxemic whilst being little or not tachypneic with relatively mild dyspnea, especially during the initial phase of Covid-19 ARF. Although we found no evidence that RR was related to subsequent HFNO failure, a prospective cohort study suggested that RR between 30 min and H6 after HFNO initiation was a simpler and more accurate parameter than ROX index to predict HFNO failure in Covid-19 ARF patients [26]. At the same time, differences could also depend on the FiO_2_ used. Indeed, we titrated FiO_2_ lower than reported for non-Covid-19 populations despite similar algorithms [18, 19]. Furthermore, a cohort study on 2040 Covid-19 ARF patients reported a better performance of SpO_2_/FiO_2_ ratio than ROX index in terms of the AUROC to predict HFNO failure at admission to emergency department [32]. The ROX index value could also be influenced by the level of gas flow rate set with HFNO [33]. In fact, the risk of intubation may not only depend on respiratory conditions and HFNO settings, but also on non-respiratory functions. A modified ROX index was thus proposed including heart rate to improve its diagnostic accuracy for hypoxemic ARF on HFNO [34]. Finally, we have shown that ROX index could be used to predict Covid-19 patients’ outcomes in the initial phase of ARF. Nevertheless, evidence is lacking regarding the extent to which ROX index cut-off values depend on populations (Covid-19 vs non Covid-19), their characteristics like the underlying cause of ARF, respiratory conditions (RR), or HFNO settings (FiO_2_, flow rate).

When covariates known at ICU admission were considered one by one, notably, the non-respiratory SOFA score appeared as a potential candidate for the parsimonious model to predict HFNO failure. Unlike others [24], we found no evidence that either this score or ROX index are essential predictors of HFNO failure. Rather, we identified SAPS II and severity of pulmonary lesions at ICU admission as potential risk factors in a parsimonious model.

This study has several limitations. First, the sample size was determined to estimate sensitivity such that its confidence interval was still meaningful but was based on reported results in a non-Covid-19 population [18]. Second, due to its observational design, intubation criteria may vary slightly between centres introducing some heterogeneity in decision-making. Nevertheless, all participating ICUs in the study used similar HFNO practices in hypoxemic ARF [6]. Consequently, the present study also provides some real-life information on HFNO practice delivered to Covid-19 ARF patients in different centres.

In conclusion, ROX index cut-off values based on Youden’s index differed from those previously described [17–19] and could be population and setting specific. The ROX index appears to have a technically acceptable but clinically low capability to discriminate between HFNO failures and successes in Covid-19 ARF patients and seems not essential in a parsimonious model to predict HNFO failure in Covid-19 ARF patients. ICU admission parameters (SAPS II and severity of pulmonary injuries at ICU admission) should also be considered because they appeared more useful than ROX index to predict HFNO outcomes in this specific population.

### Supplementary Information

**Additional file 1:** **Figure S1**. Receiver operating characteristic curves for HFNO failure within 28 days at different times after HFNO initiation. HFNO: high-flow nasal oxygen therapy; ROC: receiver operating characteristic; H0: ROC curve at the time of HFNO initiation; H12:12 hours after HFNO initiation; H18: 18 hours after HFNO initiation; H24:24 hours after HFNO initiation. **Figure S2**. Rox index performance to predict the risk of HFNO failure at different times after HFNO initiation. HFNO: high-flow nasal oxygen therapy; red line gives proportion of patients in the HFNO failure group with a ROX index ≤ a chosen cut-off value; black line gives proportion of patients in the HFNO success group with a ROX index ≤ a chosen cut-off value. For example: at H6, using a Rox index of ≤8.50 as cut-off would identify 90% of patients with HFNO failure after H6, whereas this cut-off would identify only 38% of patients with HFNO success after H6, avoiding intubation. **Figure S3**. Incidence of HFNO failure within 7 days after HFNO initiation. HFNO: high-flow nasal oxygen therapy. **Table S4**. Conditions of intubation and clinical respiratory parameters in all intubated patients and according to hemodynamic status. HFNO: high-flow nasal oxygen therapy; FiO_2_: fraction of inspired oxygen; SpO_2_: pulse oxygen saturation; RR: respiratory rate; values are expressed as n (%) or median (Q1-Q3). **Table S5**. Rox index at H0, HFNO outcome and duration according to each ICU center. HFNO: high-flow nasal oxygen therapy; ICU: intensive care unit; values are expressed as n (%) or median (Q1-Q3); *= logistic regression unless stated otherwise; **= Kruskall-Wallis’s test.

## Data Availability

The datasets used and/or analysed during the current study are available from the corresponding author on reasonable request.

## Abbreviations

ABG: Arterial blood gas
AIC: Akaike’s information criterion
ARDS: Acute respiratory distress syndrome
ARF: Acute respiratory failure
AUROC: Area under the ROC curve
Covid-19: Coronavirus disease emerged in 2019
FiO_2_: Fraction of inspired oxygen
HFNO: High-flow nasal oxygenation
ICU: Intensive care unit
MV: Invasive mechanical ventilation
NIV: Non-invasive ventilation
ROC: Receiver operating characteristic
ROX index: Ratio of SpO_2_/FiO_2_/RR
RR: Respiratory rate
SAPS II: Simplified Acute Physiologic Score II
SARS-CoV-2: Severe acute respiratory syndrome coronavirus 2
SOFA: Sequential Organ Failure Assessment
SpO_2_: Pulse oximetry
