# Supplementary material for: ROX index performance to predict high-flow nasal oxygen outcome in Covid-19 related hypoxemic acute respiratory failure
Source: Ann Intensive Care. 2024 Jan 18;14:13. doi: 10.1186/s13613-023-01226-6 (PMC10796865; doi:10.1186/s13613-023-01226-6)
Supplement: Supplementary file 1 — Additional file 1: Figure S1. Receiver operating characteristic curves for HFNO failure within 28 days at different times after HFNO initiation. HFNO: high-flow nasal oxygen therapy; ROC: receiver operating characteristic; H0: ROC curve at the time of HFNO initiation; H12:12 hours after HFNO initiation; H18: 18 hours after HFNO initiation; H24:24 hours after HFNO initiation. Figure S2. Rox index performance to predict the risk of HFNO failure at different times after HFNO initiation. HFNO: high-flow nasal oxygen therapy; red line gives proportion of patients in the HFNO failure group with a ROX index ≤ a chosen cut-off value; black line gives proportion of patients in the HFNO success group with a ROX index ≤ a chosen cut-off value. For example: at H6, using a Rox index of ≤8.50 as cut-off would identify 90% of patients with HFNO failure after H6, whereas this cut-off would identify only 38% of patients with HFNO success after H6, avoiding intubation. Figure S3. Incidence of HFNO failure within 7 days after HFNO initiation. HFNO: high-flow nasal oxygen therapy. Table S4. Conditions of intubation and clinical respiratory parameters in all intubated patients and according to hemodynamic status. HFNO: high-flow nasal oxygen therapy; FiO2: fraction of inspired oxygen; SpO2: pulse oxygen saturation; RR: respiratory rate; values are expressed as n (%) or median (Q1-Q3). Table S5. Rox index at H0, HFNO outcome and duration according to each ICU center. HFNO: high-flow nasal oxygen therapy; ICU: intensive care unit; values are expressed as n (%) or median (Q1-Q3); *= logistic regression unless stated otherwise; **= Kruskall-Wallis’s test. [file 13613_2023_1226_MOESM1_ESM.doc]

**AOIC-D-23-00589 :** Revision 2

**Electronic supplementary material (ESM)**

**ROX index performance to predict high-flow nasal oxygen outcome in Covid-19 related hypoxemic acute respiratory failure**

Christophe Girault M.D.^1^, Michael Bubenheim, Ph.D. ^2^, Déborah Boyer, M.D. ^3^, Pierre-Louis Declercq M.D. ^4^, Guillaume Schnell M.D. ^5^, Philippe Gouin M.D.^6^, Jean-Baptiste Michot M.D. ^7^, Dorothée Carpentier, M.D. ^3^, Steven Grangé, M.D. ^3^, Gaëtan Béduneau, M.D. ^1^, Fabienne Tamion M.D., Ph.D. ^8^

**Figure and table legends**

**ESM 1 (Figure).** **Receiver operating characteristic curves for HFNO failure within 28 days at different times after HFNO initiation**

HFNO: high-flow nasal oxygen therapy; ROC: receiver operating characteristic**;** H0: ROC curve at the time of HFNO initiation; H12:12 hours after HFNO initiation; H18: 18 hours after HFNO initiation; H24:24 hours after HFNO initiation

**ESM 2 (Figure).** **Rox index performance to predict the risk of HFNO failure at different times after HFNO initiation**

HFNO: high-flow nasal oxygen therapy; red line gives proportion of patients in the HFNO failure group with a ROX index ≤ a chosen cut-off value; black line gives proportion of patients in the HFNO success group with a ROX index ≤ a chosen cut-off value.

For example: at H6, using a Rox index of ≤8.50 as cut-off would identify 90% of patients with HFNO failure after H6, whereas this cut-off would identify only 38% of patients with HFNO success after H6, avoiding intubation

**ESM 3 (Figure). Incidence of HFNO failure within 7 days after HFNO initiation**

HFNO: high-flow nasal oxygen therapy

**ESM 4 (Table). Conditions of intubation and clinical respiratory parameters in all intubated patients and according to hemodynamic status**

HFNO: high-flow nasal oxygen therapy; FiO_2_: fraction of inspired oxygen; SpO_2_: pulse oxygen saturation; RR: respiratory rate; values are expressed as n (%) or median (Q1-Q3)

**ESM 5 (Table). Rox index at H0, HFNO outcome and duration according to each ICU center**

HFNO: high-flow nasal oxygen therapy; ICU: intensive care unit; values are expressed as n (%) or median (Q1-Q3); *= logistic regression unless stated otherwise; **= Kruskall-Wallis’s test.

**ESM 1 (Figure). Receiver operating characteristic curves for HFNO failure within 28 days at different times after HFNO initiation**

**ESM 2 (Figure).** **Rox index performance to predict the risk of HFNO failure at different times after HFNO initiation**

**ESM 3 (Figure). Incidence of HFNO failure within 7 days after HFNO initiation**

**ESM4 (Table). Conditions of intubation and clinical respiratory parameters in all intubated patients and according to hemodynamic status**

| **Parameters**  **before intubation** | **All intubated patients**  **in HFNO failure group (n = 46)** | **Intubated patients**  **according to hemodynamic status**  **No vasopressors Vasopressors**  **(n = 35) (n= 11)** | | **p-value** |
| --- | --- | --- | --- | --- |
| Causes of intubation. n (%) |  |  |  | 0.7542 |
| Covid-19 ARF impairment | 42 (91%) | 32 (91%) | 10 (91%) |  |
| Pneumonia | 1 (2%) | 1 (3%) | - |  |
| Secondary pulmonary embolism | 2 (4%) | 1 (3%) | 1 (9%) |  |
| Other | 1 (2%) | 1 (3%) | - |  |
| Time to HFNO initiation after ICU admission (hours) | 0 (0-3) | 0 (0-3) | 0 (0-2) | 0.3044 |
| Time to intubation after symptom onset (days) | 10 (8-15) | 10 (8-15) | 14 (5-16) | 0.6775 |
| Time to intubation after ICU admission (hours) | 29 (11-70 ) | 38 (20-71) | 6 (3-14) | **0.0017** |
| Time to intubation after HFNO initiation (hours) | 22 (9-60) | 34 (15-67) | 6 (3-14) | **0.0010** |
| HFNO conditions |  |  |  |  |
| Flow rate (L/min) | 50 (45-50 ) | 50 (45-50) | 50 (45-60) | 0.5053 |
| FiO_2_ (%) | 80 (70-100 ) | 85 (80-100) | 70 (60-100) | 0.1098 |
| SpO_2_ (%) | 92 (89-95 ) | 92 (89-93) | 95 (86-98) | 0.1151 |
| SpO_2_ /FiO_2_ (%) | 108 (91-120) | 108 (91-119) | 114 (97-165) | 0.1845 |
| RR (cycles/min) | 27 (24 -33 ) | 30 (24-34) | 26 (23-27) | 0.1968 |
| Rox index (SpO_2_ /FiO_2_ /RR) | 3.83 (3.14 -4.70 ) | 3.83 (3.03-4.60) | 4.40 (3.19-7.17) | 0.1456 |
| Arterial blood gas (n = 35) |  |  |  |  |
| PaO_2_ (mmHg) | 63 (55-73 ) | 66 (52-73) | 55 (55-63) | 0.6653 |
| PaO_2_ /FiO_2_ (mmHg) | 73 (61-94) | 68 (60-94) | 73 (63-91) | 0.3981 |
| SaO_2_ (%) | 94 (90-95) | 92 (88-95) | 94 (92-95) | 0.2710 |
| pH | 7.45 (7.43-7.49) | 7.45 (7.42-7.49) | 7.45 (7.44-7.48) | 0.4417 |
| PaCO_2_ (mmHg) | 34 (32-40) | 35 (32-40) | 31 (29-33) | **0.0262** |
| HCO3^-^ (mmol/L) | 24.4 (23.3-27.1) | 25 (23.4-27.8) | 23.8 (19.9-24.2) | 0.1687 |

Legend : HFNO: high-flow nasal oxygen therapy; FiO_2_ : fraction of inspired oxygen; SpO_2_ : pulse oxygen saturation; RR : respiratory rate; values are expressed as n (%) or median (Q1-Q3).

**ESM 5 (Table). Rox index at H0, HFNO outcome and duration according to each ICU center**

|  | **Parameters in each center** | **Overall population** | **HFNO success group** | **HFNO failure group** | **p-value*** |  |
| --- | --- | --- | --- | --- | --- | --- |
|  |  |  |  |  |
|  | **HFNO outcome, n (%)** | **n = 99** | **n = 50** | **n = 49** | 0.1944 |  |
|  | Rouen medical ICU | 35 | 20 (40%) | 15 (31%) |  |  |
|  | Rouen surgical ICU | 18 | 12 (24%) | 6 (12%) |  |  |
|  | Elbeuf medico-surgical ICU | 9 | 4 (8%) | 5 (10%) |  |  |
|  | Dieppe medico-surgical ICU | 19 | 9 (18%) | 10 (20%) |  |  |
|  | Le Havre medico-surgical ICU | 18 | 5 (10%) | 13 (27%) |  |  |
|  | **Rox index at H0** | **n = 99** | **n = 50** | **n = 49** | 0.0531 ****** |  |
|  | Rouen medical ICU | 35 | 20 8.84 (6.86-10.33) | 15 6.92 (5.17-10.11) |  |  |
|  | Rouen surgical ICU | 18 | 12 6.71 (5.46-9.90) | 6 4.56 (3.79-6.03) |  |  |
|  | Elbeuf medico-surgical ICU | 9 | 4 4.66 (3.19-6.23) | 5 5.52 (3.91-5.54) |  |  |
|  | Dieppe medico-surgical ICU | 19 | 9 9.61 (6.74-13.20) | 10 5.11 (4.55-6.46) |  |  |
|  | Le Havre medico-surgical ICU | 18 | 5 7.68 (6.40-9.29) | 13 6.20 (5.64-7.31) |  |  |
|  | **Time to intubation after HFNO initiation (hours)** | **n = 46** |  | **n = 46** | 0.1771 ****** |  |
|  | Rouen medical ICU | 12 | - | 6 (3-12) |  |  |
|  | Rouen surgical ICU | 6 | - | 34 (11-114) |  |  |
|  | Elbeuf medico-surgical ICU | 5 | - | 30 (22-45) |  |  |
|  | Dieppe medico-surgical ICU | 10 | - | 22 (9-61) |  |  |
|  | Le Havre medico-surgical ICU | 13 | - | 36 (21-52) |  | |

Legend : HFNO: high-flow nasal oxygen therapy; ICU: intensive care unit; values are expressed as n (%) or median (Q1-Q3); *= logistic regression unless stated otherwise; **= Kruskall-Wallis’s test
